# A Red Eye in an Elderly Patient: A Case Report Highlighting the Importance of Urgent Diagnosis

**DOI:** 10.7759/cureus.100117

**Published:** 2025-12-26

**Authors:** Deepa Ragesh Panikkath

**Affiliations:** 1 Rheumatology, David Geffen School of Medicine, University of California, Los Angeles, Los Angeles, USA

**Keywords:** acute scleritis, anca-associated vasculitis, c-anca/proteinase 3-positive granulomatosis with polyangiitis, gpa, granulomatosis with polyangiitis (gpa), inflammatory eye diseases, pr3 antibody, scleritis, systemic vasculitis

## Abstract

Anti-neutrophil cytoplasmic antibody (ANCA)-associated vasculitides are a distinct group of autoimmune disorders characterized by necrotizing inflammation of small blood vessels. Three distinct clinical entities are described, including eosinophilic granulomatosis with polyangiitis (EGPA), granulomatosis with polyangiitis (GPA), and microscopic polyangiitis (MPA). GPA often presents as a multi-system inflammatory disease frequently involving the sinuses, kidney, and lung tissue. Ophthalmologic involvement has been described in GPA and is highly important to recognize, especially if presenting as the initial symptom of the disease. Prompt diagnosis and treatment are essential, given the significant morbidity and mortality associated with GPA.

## Introduction

Anti-neutrophil cytoplasmic antibody (ANCA)-associated vasculitides (AAVs) consist of a group of autoimmune diseases characterized by small- to medium-vessel wall inflammation [1]. Granulomatosis with polyangiitis (GPA) is one of the subtypes and is typically characterized by granulomatous inflammation, necrosis, and vasculitis [1]. Presentations in GPA patients can vary from a limited subtype, in which usually a single organ is affected, to a generalized multisystem organ involvement. The classic triad consists of necrotizing upper and lower respiratory tract granulomas, small vessel vasculitis, and necrotizing glomerulonephritis [1,2].

The diagnosis is based on clinical findings, serology, imaging, and confirmation by tissue biopsy whenever feasible. GPA is typically associated with cytoplasmic ANCA (c-ANCA) antibodies targeting proteinase 3 in 60-95% of patients [3]. In 10% of cases, antibodies against myeloperoxidase (MPO) are positive, and rarely, patients can also present with ANCA-negative disease [3]. These antibodies trigger an inflammatory cascade involving neutrophils and the complement pathway, resulting in necrotizing tissue inflammation and granuloma formation [4]. Ocular involvement is seen in approximately 30-50% of patients with GPA and can involve any part of the eye [5,6]. Initial manifestation can be scleritis, and different kinds have been described, such as nodular, diffuse, or necrotizing [7]. Other ocular manifestations include peripheral ulcerative keratitis, retinitis, and rarely retinal necrosis [7].

This case report illustrates an ocular presentation of GPA in a 74-year-old female in the absence of any systemic involvement. The case highlights the high degree of clinical suspicion needed in such scenarios when disease occurs in a limited form.

## Case presentation

A 74-year-old female with a history of systemic arterial hypertension developed new onset pain and redness of the left eye of six months' duration. Initially, she was diagnosed to have episcleritis and was treated with prednisolone eye drops with no relief. She had worsening eye pain, redness, and developed left-sided headache and facial pain that resulted in an emergency room visit. She did not have any accompanying vision issues.

During the ER visit, she was noted to have mild eyelid swelling without erythema of the upper and lower eyelids. There was marked hyperemia and engorged blood vessels on the nasal aspect of her left bulbar conjunctiva. On slit-lamp exam, no hypopyon or hyphema was noted. She had a challenging slit-lamp exam due to her inability to fully cooperate and open her eye. On fluorescein stain, no obvious uptake was noted in the cornea, and no corneal ulceration was seen. Intraocular pressures measured were within normal limits.

**Figure 1:**
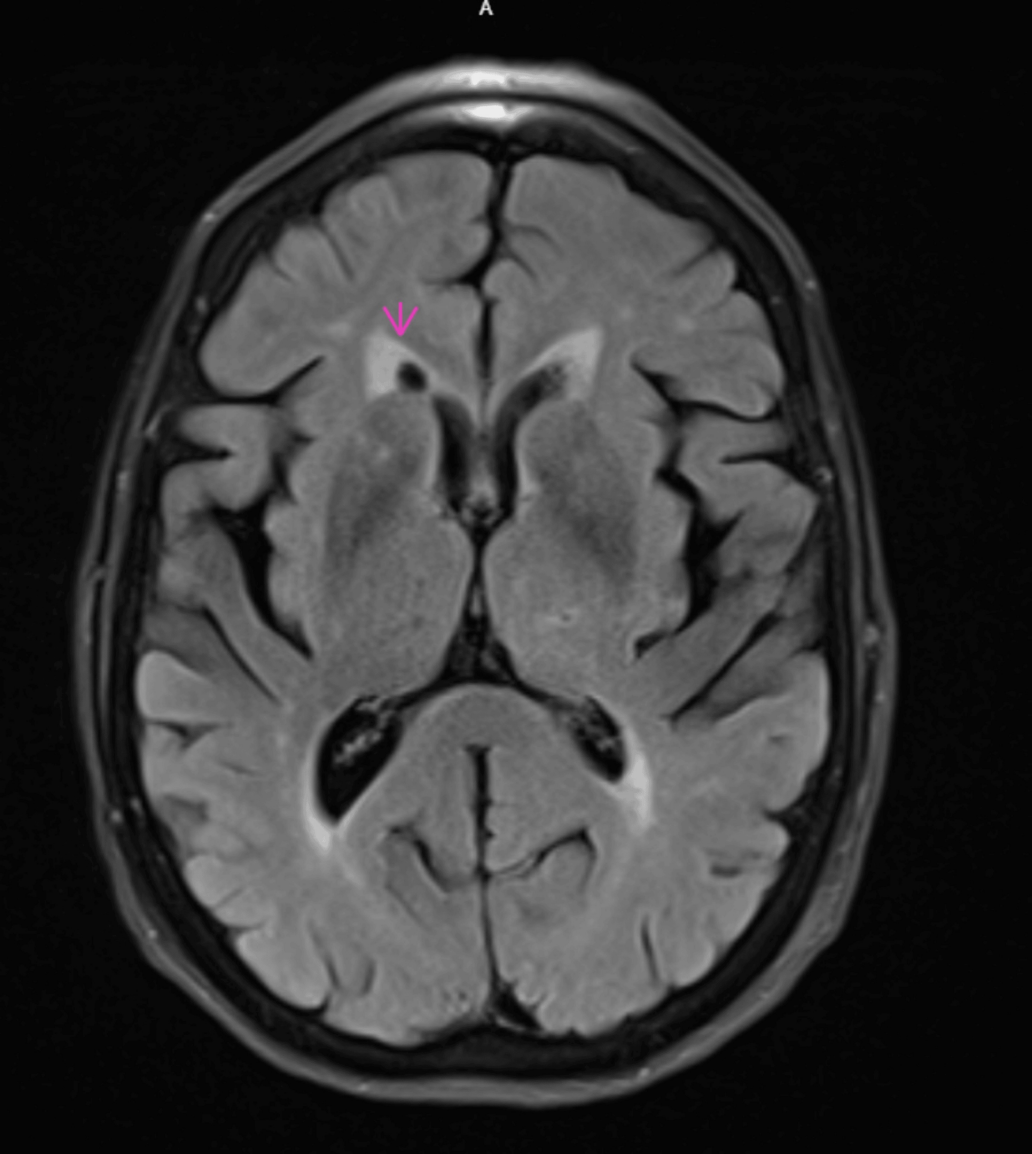
Axial T2 FLAIR MRI of the brain without contrast. FLAIR: fluid-attenuated inversion recovery.

Laboratory workup for complete blood counts and liver and kidney function tests was unremarkable. Temporal arteritis was suspected, and inflammatory markers, including erythrocyte sedimentation rate (ESR) and C-reactive protein (CRP), were within normal limits. A magnetic resonance imaging (MRI) of the brain was done, which was unremarkable for any acute intracranial abnormality, and showed no evidence of mass, acute ischemia, or hemorrhage. It revealed a small old lacunar infarct in the right frontal gangliocapsular region (Figure 1). She was discharged from the ER and subsequently evaluated by ophthalmology. She was diagnosed with anterior scleritis and started on oral corticosteroids at an initial dosage of 40 mg of oral prednisone daily.

Extensive blood work was done, including infection screening, resulting in negative treponemal studies, hepatitis panel, and QuantiFERON tests. Angiotensin-converting enzyme (ACE) levels were normal. Autoimmune labs were negative for antinuclear antibodies (ANA), extractable nuclear antigen (ENA), including anti-Ro/SSA and anti-La/SSB antibodies, rheumatoid factor (RF), anti-cyclic citrullinated peptide (anti-CCP) antibodies, and human leukocyte antigen B27 (HLA-B27) gene. Additional autoimmune workup was done, which resulted positive for c-ANCA at a titer of 1:80 and PR3 antibody. MPO antibody and perinuclear-ANCA titer were negative (Tables 1, 2).

**Table 1:** Laboratory tests. WBC: white blood cells; eGFR: estimated glomerular filtration rate; ALT: alanine aminotransferase; AST: aspartate aminotransferase; ACE: angiotensin-converting enzyme; Ab: antibodies; Ag: antigen.

Laboratory test	Patient value	Reference range & units
WBC	4.5	3.8 - 11.8 K/uL
Hemoglobin	12.2	10.9 - 16.0 gm/dL
Hematocrit	36.2	32.0 - 46.0%
Platelet	130	150 - 450 K/ul
Sedimentation rate	7	0 - 30 mm/hr
C-reactive protein	0.37	0.00 - 0.50 mg/dl
Blood urea nitrogen	18	7 - 19 mg/dl
Creatinine	0.81	0.00 - 0.50 mg/dl
eGFR	76	>=90 ml/min/1.73 m^2^
Total protein	6.3	6.4 - 8.3 gm/dl
Albumin	3.8	3.5 - 5.0 gm/dl
Globulin	2.5	2.0 - 4.0 gm/dl
Alkaline phosphatase	52	40 - 150 units/L
ALT	11	0 - 55 units/L
AST	13	5 - 34 units/L
ACE	<10	16 - 85 u/l
QuantiFERON	Negative	Negative
Treponemal Ab	Nonreactive	
Hepatitis C Ab	Nonreactive	
Hepatitis B core Ab IgM	Nonreactive	
Hepatitis B surface Ag	Nonreactive	
Hepatitis B surface Ab	Nonreactive	

**Table 2:** Serology and immunology. CCP: cyclic citrullinated peptide; Ab: antibodies; HLA-B27: human leukocyte antigen B27; c-ANCA: cytoplasmic antineutrophil cytoplasmic antibody; p-ANCA: perinuclear antineutrophil cytoplasmic antibody; ANA IFA: antinuclear antibodies - indirect immunofluorescence assay.

Laboratory test	Patient value	Reference range & units
Rheumatoid factor Quant	<15.0	<=29.9
CCP IgG/IgA	4	0 - 19 unit
ANA IFA	<1: 80	<1: 80
SSA 52(Ro) Ab	7	0 - 40
SSA 60(Ro) Ab	3	0 - 40
HLA-B27	Negative	Negative
c-ANCA	1:80	<1:20
p-ANCA	Negative	<1:20
Proteinase-3 antibody	95	0 - 19 AU/ml
Myeloperoxidase antibody	0	0 - 19 AU/ml
Immunoglobulin A, serum	84	68 - 408 mg/dL
Immunoglobulin G, serum	829	768 - 1632 mg/dL
Immunoglobulin M, serum	48	35 - 263 mg/dL

In light of the positive serologies, a diagnosis of GPA was considered. She underwent screening for other organ involvement, including a sinonasal endoscopy, which was normal, with no inflammatory sinus disease or granulomas. Additional testing revealed a normal chest X-ray and normal urine analysis without evidence of proteinuria or hematuria.

She was initiated on steroid-sparing therapy with oral methotrexate, given a limited presentation with consideration of escalation in immunosuppressive treatment if inadequate or incomplete response occurs. However, she had a quick clinical response to methotrexate therapy that was initiated at a starting dose of 10 mg orally weekly and escalated up to 25 mg/week. Ophthalmology was able to taper off systemic and topical steroids completely. She now remains in clinical remission on methotrexate therapy and is being closely monitored for any systemic progression of the disease.

## Discussion

Recognition of ocular diseases in GPA is important, especially when they occur as an initial and sole manifestation, as seen in this patient. Ocular and orbital involvement is not rare and is reported to occur in up to 30-50% of patients, and in 6-18%, it can be the first presentation of the disease [8]. Ocular disease has also been shown to predict relapse or progression of disease in some cases [2].

In a retrospective, cross-sectional, observational study of 63 patients with GPA, scleritis was the most common ocular manifestation (36%), followed by retro-orbital pseudotumor (23%) and episcleritis (13%) [8]. Both anterior and posterior scleritis have been reported in GPA patients [8]. The exact mechanism of pathogenesis is unknown, and scleral collagen is considered to be the target for autoantibodies [7]. Scleritis can be complicated by necrotizing inflammation and globe perforation, leading to vision loss [8]. Other vision-threatening manifestations in GPA include compressive optic neuropathy and retinal and optic nerve vasculitis [9].

The diagnosis is based on clinical presentation, serological testing for ANCA, imaging, and tissue biopsy if feasible. ANCA testing can be considered as a screening tool, given that the majority of AAV patients with ocular disease are ANCA positive [10]. Serological positivity to c-ANCA/PR3 antibodies is one of the domains included in the 2022 American College of Rheumatology/European Alliance of Associations for Rheumatology (ACR/EULAR) classification criteria for GPA [11]. This patient did not have the classic features of GPA, such as nasal/paranasal, lung, and kidney involvement, all of which are included in the 2022 ACR/EULAR classification criteria for GPA [11]. She presented with a limited form of GPA with isolated ocular involvement.

Treatment decisions are made based on the patient’s overall disease presentation. When limited to the eye, mild ocular manifestations such as episcleritis, conjunctivitis, and mild anterior uveitis may respond to topical therapy alone. Systemic corticosteroid therapy and additional cytotoxic systemic agents such as cyclophosphamide, azathioprine, methotrexate, and rituximab are often used for the management of severe and vision-threatening ocular disease [2]. Local surgical management may be necessary for orbital decompression or debulking in cases with orbital granulomas.

In general, response to therapy is good with clinical remission achieved in over 90% of the patients with ANCA-associated ocular disease [10]. Relapse can occur in any presentation of ocular disease and can be independent without flare in other organ systems. The type of ANCA vasculitis and serology are poor predictors of relapse [10]. Prognosis varies based on the severity and chronicity of presentation. Permanent visual loss or blindness may occur in up to 8-37% of patients [9].

## Conclusions

Diagnosis of GPA in patients presenting solely with ocular involvement can be difficult. As seen in this case, a high degree of clinical suspicion is needed as the disease presented without the classic triad of sinus, lung, and renal involvement. Delay in diagnosis can happen in such cases, which can be detrimental, resulting in complications that can lead to permanent vision loss. Hence, screening for ANCA serology in patients with scleritis can be valuable as it may be the initial or an isolated manifestation of the disease. Multispecialty collaboration between ophthalmology and rheumatology is hence critical for optimal management of ophthalmic disease in GPA.
